# CARE PARTNERS’ ENGAGEMENT IN PREVENTING FALLS FOR COMMUNITY-DWELLING OLDER PEOPLE WITH DEMENTIA

**DOI:** 10.1093/geroni/igad104.0280

**Published:** 2023-12-21

**Authors:** Yuanjin Zhou, Clara Berridge, Nancy Hooyman, Elizabeth Phelan

**Affiliations:** University of Texas at Austin, Austin, Texas, United States; University of Washington, Seattle, Washington, United States; University of Washington, Seattle, Washington, United States; University Of Washington, School Of Medicine, Seattle, Washington, United States

## Abstract

Care partners adopt various behaviors to manage fall risk for community-dwelling older people with dementia (cd-OPWD). It is unknown if these behaviors reduce falls for cd-OPWD at different levels of fall risk. We linked the data from the 2015 and 2016 National Health and Aging Trends Study (NHATS) and the 2015 National Study of Caregiving (NSOC) (cd-OPWD n=365, 89% aged 75+) to explore this question. The outcome was no fall, one fall, and multiple falls in 2016. We used the same variable in 2015 to indicate fall risk at baseline. We identified 28 items from NSOC to represent dementia care partners’ fall risk management (FRM) behaviors based on existing literature and expert review. Using exploratory factor analysis, we identified five categories of FRM behaviors: mobility and safety assistance, health management, accommodations, medical services coordination, and social services coordination. We performed multinomial logistic regression to investigate associations between care partners’ FRM behaviors in 2015 and cd-OPWD’ falls in 2016 stratified by cd-OPWD’s history of falls in 2015, adjusting for selected covariates. For cd-OPWD who had no fall in 2015, mobility and safety assistance was associated with a higher risk of having one fall in 2016; for those who had one fall in 2015, medical services coordination was associated with a lower risk of multiple falls; for those who had multiple falls in 2015, social services coordination was associated with a lower risk of multiple falls. Future research may target care partners’ different FRM behaviors based on cd-OPWD’s history of falls.

